# 

**Published:** 1873-12-01

**Authors:** 


Our Publication Office.—We would call the attention of our subscribers and friends to the fact that the office of The Medical Examiner has been removed to No. 65 Randolph street, N. W. cor. State, where all communications for the editors should be addressed.

